# Green Extraction Methods Applied to the Brown Macroalga *Saccharina latissima*: Assessing Yield, Total Phenolics, Phlorotannins and Antioxidant Capacity

**DOI:** 10.3390/foods14061017

**Published:** 2025-03-17

**Authors:** Jonas da Silva, Luana Cristina dos Santos, Elena Ibañez, Sandra Regina Salvador Ferreira

**Affiliations:** 1 Chemical Engineering and Food Engineering Department, Federal University of Santa Catarina, EQA, CTC-UFSC, Florianópolis 88040-900, SC, Brazil; jonasjmfamilia@gmail.com; 2 Foodomics Laboratory, Department of Bioactivity and Food Analysis, CIAL, CSIC-UAM, Nicolas Cabrera 9, 28049 Madrid, Spain; luana.dsantos@csic.es

**Keywords:** brown algae, green extraction, polyphenols, phlorotannins, antioxidants, GRAS solvents

## Abstract

The brown seaweed *Saccharina latissima* is an abundant, although yet underutilized, source of natural bioactive compounds commonly found in western regions. In recent years, brown algae have garnered attention as promising sources of polyphenols, particularly phlorotannins. The recovery of these relevant components by eco-friendly and energy-efficient methods with solvents GRAS (Generally recognized as safe) contributes to minimizing environmental impact, and promotes sustainability. Pressurized liquid extraction (PLE) and microwave-assisted extraction (MAE) optimized by Box–Behnken design (BBD) were explored for this purpose. The methods were evaluated considering the process yield and the quality of the recovered extracts by phenolic and phlorotannin levels, and their antioxidant capacity was assessed by DPPH and ABTS assays. The optimized MAE techniques (80 °C, 2% EtOH/Water at 40 mL g^−1^) and PLE2 (80 °C with water) showed the highest extract yields, with increases of 65.76% and 37.36%, respectively, compared to CRE. PLE2 also achieved higher TPC and antioxidant capacity (ABTS) values by 61.88% and 80.39%, respectively. MAE (optimized) increased TPC and ABTS by 53.90% and 36.42%, respectively. Regression analysis of MAE confirmed the accuracy of the models in assessing interaction parameters (adjustment *p* < 0.05 and adequacy R^2^ > 0.86). Therefore, the study presents eco-efficient approaches for recovering phenolic compounds and antioxidants from brown algae, contributing to the valorization of these resources in the industry and enhancing their application.

## 1. Introduction

*Saccharina latissima* (also known as “sugar kelp”) is an edible brown macroalga that can be considered a sustainable source of natural products with potential health benefits [1]. For instance, among the molecules only found in brown macroalgae, phlorotannin is a polyphenol with unique structure and noticeable bioactivities, as corroborated by numerous applications in food and cosmetic products, mainly associated with its antioxidant properties [2].

Polyphenols from plants and seaweed can play an important metabolic role as antioxidant components which can scavenge free radicals and reactive oxygen species, besides many other biological activities (e.g., anti-diabetic and anti-inflammatory, among others) [3]. In the human body, oxidative pathways are mainly linked to reactive oxygen species (ROS), i.e., reactive molecules that cause broad oxidative cell damage, and consequently, these pathways are related to diseases such as atherosclerosis, cancer and even neurological disorders. Therefore, phlorotannin-rich extracts, with high antioxidant potential, are potential candidates to work against such degenerative processes [4].

The antioxidant capacity, as well as the polyphenols content present in brown algae, is strictly related to the type of alga, region of cropping, and also to the extraction method applied. Emerging and sustainable extraction methods for phlorotannins are currently preferred as they are more environmentally friendly than conventional methods [5,6]. In addition, they can replace toxic organic solvents with greener solvents, and/or accelerate the mass transfer extraction kinetics by means of intensification of extra variables (e.g., cavitation, pressure, wave frequency). Currently, there are few works describing phlorotannin extraction from *Saccharina latissima*, by methods that can take up to 20 h [7].

Among the emerging extraction techniques, microwave-assisted extraction (MAE) and pressurized liquid extraction (PLE) have been successfully described for application in marine brown macroalgae, mainly focusing on antioxidant extract recovery [8,9]. MAE and PLE can be conducted using green solvents and are associated with an intensification parameter (microwaves for MAE and high pressure for PLE), which improves the process yield, and reduces the extraction time and the solvent and energy consumption. Briefly, microwave of frequency up to 300 GHz are used for heating the solvent and the extractable material, while high pressure allows for fast extraction due to the high temperatures achieved (above the boiling point) of the selected solvent, which is maintained in a liquid state [10]. The solvent selection for both extraction techniques generally depends on the molecular class of the target compounds, with water or aqueous ethanol mixtures being preferred for the recovery of phlorotannins of low to intermediate molecular masses due to their solute–solvent affinity, environmental safety (GRAS solvents), and ease of handling due to their low viscosity [9,11,12]. In contrast, bound phlorotannins generally require alkaline extraction. It is also important to adequately define the process parameters which validate the extraction of polyphenols and antioxidant compounds, in order to optimize the operation and reduce costs.

Considering that the extraction of valuable bioactive compounds from brown macroalgae is of industrial interest, the objective of the present study is the recovery of polyphenols (phenolics and phlorotannins) and other antioxidant compounds from *Saccharina latissima* using alternative MAE and PLE methods with GRAS solvents. These novel extraction approaches were compared with a conventional extraction based on solvent reflux using the same solvents. The biological potential of the extracts were observed through in vitro assays. This innovative approach highlights the relevance of extracting different bioactive fractions from *Saccharina latissima*.

## 2. Materials and Methods

### 2.1. Proximate Composition of the Raw Material

Samples of the macroalgae *Saccharina latissima* were kindly donated by Porto-Muiños S.L., located in Cerceda, A Coruña, Spain on 24 January 2022, and were received vacuum-packed, freeze-dried and milled. Samples were first characterized in terms of moisture content by the gravimetric method in a Kern DAB 100-3 moisture analyzer from KERN and SOHN (Balingen, Germany), while protein, ash, and lipid content were obtained according to official methods 970.22, 972.15, and 963.15 from the Association of Official Analytical Chemists [13], respectively.

### 2.2. Chemicals and Reagents

Analytical-grade Folin–Ciocalteu reagent, gallic acid, phloroglucinol dihydrate, DMBA (2,4-dimethoxybenzaldehyde), DPPH (2,2-diphenyl−1-picrylhydrazyl), ABTS (2,2′-azinobis-3-ethylbenzothiazoline-6-sulfonic-acid), and Trolox (6-hydroxy-2,5,7,8-tetra-methylchroman-2 carboxylic acid) were purchased from Sigma-Aldrich (Darmstadt, Germany). Sodium carbonate, sodium hydroxide, and acetic acid were purchased from Neon (Sao Paulo, Brazil). Hydrochloric acid, and potassium persulfate were purchased from Êxodo (Feira de Santana, Brazil). 2,2′-Azobis(2-methylpropionamidine) dihydrochloride (AAPH) was purchased from TCI Chemicals (Tokyo, Japan), while monopotassium phosphate (KH_2_PO_4_), fluorescein sodium salt, and ascorbic acid were purchased from Sigma-Aldrich (Madrid, Spain). Ethanol P.A. and distilled water were used in the preparation of solutions.

### 2.3. Extraction Processes

#### 2.3.1. Conventional Reflux Extraction (CRE)

CRE was conducted according to classical methods, following the method number 920.39C with modifications [14]. For the conventional extractions, the solvents used were water, ethanol/water mixtures (50%), and ethanol (100% P.A.). An amount of 180 mL of solvent was placed inside the conventional apparatus, in a paper cartridge, along with 5 g of dried sample. The extraction was carried out with solvent reflux (boiling solvent) for up to 12 cycles over 6 h.

#### 2.3.2. Microwave-Assisted Extraction (MAE)

MAE was performed in a microwave oven (Monowave 300—Anton Paar GmbH, Graz, Austria) with a maximum of 850 W and constant agitation of 1000 rpm. A flask, model G30, was used for the extraction with a magnetic stirrer. The assays were carried out based on a Box–Behnken design (BBD) with three independent variables and three levels: (1) temperature (T) at 40, 60 and 80 °C; (2) ethanol/water concentration (EtOH) at 0%, 50% and 100% absolute *v*/*v*; and (3) liquid/solid ratio (L/S) at 10:1, 25:1 and 40:1 mL g^−1^.

Previous to MAE evaluation at different variable conditions, the kinetics curve was constructed for MAE using the central point parameters (Section 2.3.4). The MAE kinetics allow for definition of the extraction time, after reaching the diffusional period, with almost null extraction rate [15]. The MAE assays were conducted following the BBD experimental plan, with the recovered extract samples filtered, rotary evaporated and freeze-dried, and stored at −18 °C until further analysis. The experimental design was analyzed using Statistica 13.5 software (TibCo, Santa Clara, CA, USA), and a desirability function was applied to define the optimal condition of multiple responses. The second-order polynomial model (quadratic model) for the response surface analysis is shown as follows (Equation (1)).

(1) $$R=\propto_{0}+\sum \propto_{i}\beta_{i}+\sum \propto_{ii}\beta_{i}^{2}+\sum \propto_{ij}\beta_{i}\beta_{j}$$

where *R* is the response variable, *α*_0_ is the constant, *α_i_* is the linear effect., *α_ii_* is the quadratic effect, and *α_ij_* is the interaction effect, while *β_i_* and *β_j_* are the independent variables.

#### 2.3.3. Pressurized Liquid Extraction (PLE)

PLE was conducted in continuous mode utilizing a self-assembled apparatus outlined by Gonçalves Rodrigues et al. [16]. Previously, a kinetics study was carried out to determine the extraction time (Section 2.3.4), defined from the extraction curve after reaching the diffusional period, with low extraction rate [15]. After the definition of extraction time, a group of PLE assays with varying process conditions was conducted in triplicate. The extractions were performed under the following conditions: 100% EtOH absolute and 40 °C; 50% EtOH and 40 °C; and using water with temperatures of 40, 80 and 120 °C. Briefly, 3 g of dried powdered sample and 40 g of glass beads were placed inside the stainless-steel extraction vessel of 90 mL capacity (25 mm inner diameter and 180 mm height). A pump (Waters, model 515, Milford, CT, USA), drove the solvent into the extraction cell (4 mL min^−1^), and a needle valve regulated the pressure at 10 MPa (HiP, model 20–11LF4, NFA, Chicago, IL, USA). The recovered extracts were dried to remove the solvent using a rotary evaporator or freezer dryer, and then were stored at −18 °C in absence of light until further analysis.

#### 2.3.4. Kinetics Study for the Alternative Extraction Methods

Microwave-assisted extraction (MAE) and pressurized liquid extraction (PLE) were the alternative extraction procedures used. A kinetics study was carried out, before MAE and PLE optimization assays, in order to determine the extraction time by evaluating the mass transfer mechanisms from the kinetics extraction curve. The MAE kinetics curve was obtained for the conditions of 60 °C, 50% EtOH, and 25 mL g^−1^, the midpoint condition for the group of MAE assays (Section 2.3.2). The PLE kinetics curves were obtained at constant pressure (10 MPa) and flow rate (4 mL min^−1^), and at temperatures of 40 and 80 °C (midpoint, related to PLE conditions: Section 2.3.3), with 50% EtOH and water, respectively.

The Piecewise function (Equation (2)) used was composed of three straight lines, corresponding to the mass transfer mechanisms that represent the three extraction rate periods: Constant extraction rate (CER), Falling extraction rate (FER) and Diffusional controlled period (DCR) [17]. The equation system (1) was adjusted to the experimental data to define the appropriate extraction time for maximum yield and minimum solvent and energy consumption.

(2) $$y=\left\{\begin{array}{c} a_{1}+k_{1}x\;\left(x<x_{i1}\right)\;\\ y_{i1}+k_{2}\left(x<x_{i1}\right)\;\left(x_{i1}\leq x{<x}_{i2}\right)\\ y_{i2}+k_{3}\left(x<x_{i2}\right)\;\left(x\geq x_{i2}\right)\; \end{array}\right.$$

This resulted in the following equations (Equations (3) and (4)):

(3) $$y_{i1}=a_{1}+k_{1}x_{i1}\;$$

(4) $${\;y}_{i2}=y_{i1}+k_{2}\left(x_{i2}-x_{i1}\right)$$

where *a*_1_ = Intercept, *k*_1_ = Slope, *x_i_*_1_ = Intersection, *k*_2_ = Slope, *x_i_*_2_ = Intersection, *k*_3_ = Slope. (*x* = time of extraction, and *y* = yield extraction).

#### 2.3.5. Extraction Yield (*E_y_*)

*E_y_* was calculated as the percentage of the ratio between the extracted mass (*m_e_*) and the dry sample mass (*m_d_*) according to Equation (5).

(5) $$E_{y=}\frac{m_{e}}{m_{d}}\times 100$$

### 2.4. Chemical Analysis

#### 2.4.1. Total Phenolics Content (TPC) and UHPLC-q-TOF-MS/MS

TPC was determined according to the method of Singleton, Orthofer, and Lamuela-Raventós [18]. A standard gallic acid curve (0 at 1 mg mL^−1^) was prepared, presenting an R^2^ value of 0.9968. Solutions of the extract samples recovered by CRE, MAE, and PLE were prepared. The reaction was carried out with 10 µL of extract, 50 µL of Folin–Ciocalteau reagent, 150 µL of 20% sodium carbonate, and 790 µL of distilled water. The samples were incubated in darkness at room temperature. After 2 h incubation, absorbance was measured on a spectrophotometer (Agilent, Epoch-BioTek, Santa Clara, CA, USA) at 760 nm. The results were expressed in milligrams of gallic acid equivalent (GAE) per gram of dried extract (mg GAE g^−1^). Analyses were carried out in triplicate.

Ultra-high performance liquid chromatography (Agilent 1290 UHPLC system) coupled to a high-resolution quadrupole time-of-flight mass spectrometer (qTOF-MS/MS) was performed in this work to attempt phenolic identification for selected extract samples obtained under optimized conditions of MAE and ideal conditions of PLE (Section 3.2 and Section 3.3), following the method described in detail elsewhere [19].

#### 2.4.2. Total Phlorotannins Content (TPTs)

The total phlorotannins content from the extract samples obtained by CRE, MAE, and PLE was determined according to the DMBA colorimetric test [20]. The DMBA solution was prepared with a 1:1 ratio of (DMBA 2%):(HCl 6%), and both solutions were prepared in acetic acid. A standard curve using phloroglucinol dihydrate (0.00098 at 0.0625 mg mL^−1^) was prepared, with an R^2^ equal to 0.999. A total of 50 μL of diluted extract was mixed with 250 μL of DMBA solution, and the reaction was conducted at room temperature for 60 min in the dark. After this period, absorbance was measured on a spectrophotometer (Agilent, Epoch-BioTek, USA) at 515 nm. The results were expressed in milligrams of phloroglucinol equivalent (PGE) per gram of dried extract (mg PGE g^−1^). Analyses were carried out in triplicate.

#### 2.4.3. DPPH Radical Scavenging Capacity

Antioxidant capacity was evaluated using the DPPH (2,2-diphenyl-1-picrylhydrazyl) free radical capture assay, according to the protocol described by Mensor et al. [21]. Trolox served as the standard to produce the calibration curve (0 at 400 μmol/L) with an R^2^ of 0.9880. Solutions of the extracts obtained by CRE, MAE, and PLE were prepared. The DPPH radical solution was added with the diluted extract and reacted for 30 min at room temperature, without light. The samples were measured using a spectrophotometer (Agilent, Epoch-BioTek, USA) at a wavelength of 517 nm. The results were reported as micromoles of Trolox Equivalent per gram of dried extract (mmol TE g^−1^). Analyses were carried out in triplicate.

#### 2.4.4. ABTS Radical Scavenging Assay

The antioxidant capacity of the extracts was also assessed following the methodology proposed by Re et al. [22]. The ABTS radical was produced by the reaction of 2.45 mM potassium persulfate and 7 mM of ABTS (2,2′-azinobis-3-ethylbenzothiazoline-6-sulfonic acid). The radical was kept in the dark and at room temperature for 16 h before use. The ABTS radical was diluted to an absorbance of 0.7 (±0.2) at 734 nm. Solutions of the extracts obtained by CRE, PLE, and MAE were prepared. Trolox was used as standard, and the calibration curve (0 at 500 μmol/L) presented a coefficient of determination (R^2^) of 0.9991. The diluted extracts were mixed with the ABTS solution and allowed to react for 45 min in the dark. After the reaction, the absorbance was read at 734 nm in a spectrophotometer (Agilent, Epoch-BioTek, USA). The results were reported in micromoles of Trolox equivalent per gram of dried extract (μmol TE g^−1^). Analyses were carried out in triplicate.

#### 2.4.5. Oxygen Radical Absorbance Capacity (ORAC)

The ORAC method reported by Ou et al. [23] was performed to quantify the reactive oxygen species scavenging capacity of the extracts of *Saccharina latissima* obtained under the ideal conditions of PLE and in optimized conditions of MAE, considering the yield and the extraction of bioactive compounds. Briefly, extract concentrations from 4 to 40 µg/mL were mixed with 100 µL of 2,2′-Azobis(2-methylpropionamidine) dihydrochloride (AAPH) at 590 mM, 100 µL of phosphate buffer at 30 mM (pH = 7.5), and 25 µL of fluorescein at 11 µM. The fluorescence kinetic reading parameters were as follows: λ excitation = 485 nm, λ emission = 530 nm at 37 °C with total kinetic reading of 1 h (with 5 min interval reading). Ascorbic acid was used as standard reference (positive control), at concentrations from 0.12 to 1.23 µg/mL. Measurements were performed in triplicate and the inhibition percentage of radicals generated from AAPH decomposition was calculated according to Equations (6) and (7).

(6) AUC=0.5×∑i=1nfif0

where *f_i_* is the fluorescence at a certain time I and *f*_0_ is the fluorescence at *t* = 0 min.

(7) $$Inhibition\left(\%\right)=\frac{{AUC}_{control}-{AUC}_{sample}}{{AUC}_{control}}\times 100$$

where *AUC_control_* and *AUC_sample_* represent the area under the curve of fluorescence decay in the absence and in the presence of the sample extract, respectively. Results were given as concentration of extract (or ascorbic acid) to achieve 50% inhibition and were represented as IC_50_ (µg of extract/mL).

### 2.5. Statistical Analysis

The results were statistically analyzed using Statistica 13.5 software (TibCo, USA) through analysis of variance (ANOVA). Multiple mean comparisons were performed utilizing the Tukey test, with a confidence level of 95%.

## 3. Results and Discussion

### 3.1. S. latissima Proximal Composition and Kinetics Curves for MAE and PLE Assays

*Saccharina latissima* was demonstrated to be a rich source of carbohydrates, followed by ash, proteins, and lipids (Table 1). In fact, most studies concerning *S. latissima* have been mainly focused on the bioactive properties of a polysaccharide fucoidan, a non-starchy form of carbohydrate [24,25,26]. Other minor components from this alga, including phlorotannins (and other polyphenols), remained poorly explored up to this time, justifying the efforts of the present work in providing novel information concerning the alga’s phenolic composition and antioxidant capacity through different mechanisms.

Kinetic studies are essential to evaluate the mass transfer mechanisms involved in the extraction process and its duration. The analysis of the extraction kinetics contributes to establishing a more cost-effective and sustainable process. Figure S1 presents the kinetics curves for MAE (at 80 °C, 2% EtOH, and 40 mL g^−1^) and PLE (at 40 °C, 50% EtOH; and at 80 °C, water). The mass transfer mechanisms were evaluated from the process kinetics, representing the extraction periods of constant extraction rate (CER), falling extraction rate (FER), and diffusion-controlled rate (DCR), as defined by supercritical processes [27], and applied for MAE [28] and PLE [28,29].

The parameters of the Piecewise function obtained by the fitting curves are listed in Table 2. The adjustment presented R^2^ values higher than 0.96, indicating the proximity of predicted and observed values. Mathematical modeling contributes to elucidation of the mass transfer mechanisms of the extraction process, helping in the definition of parameters to enhance the economic viability at industrial scale [30].

The CER period was completed in approximately 2.10, 7.73, and 7.66 min for MAE, PLE1, and PLE2, respectively. The first fitted line represents CER, where the extraction of soluble material from the surface of the solid particles takes place, characterized by the convection mechanism [31]. The partial exhaustion of the solute from the particle surfaces represents the FER period, which combines the convection and the diffusion mechanisms simultaneously, and was reached at approximately 7.10, 26.00, and 28.55 min for MAE, PLE1, and PLE2, respectively. Subsequently, the extraction rate decreases due to the exhaustion of the solute from the particle surface, controlled by diffusion (DCR period) [32]. The extraction time is defined by obtaining the DCR period, characterized by a low extraction rate ensuring complete exhaustion of the solute. This analysis resulted in extraction times defined as 8, 30, and 30 min for MAE, PLE1, and PLE2, respectively.

### 3.2. MAE Process

The BBD was used to optimize the MAE independent variables (T °C, EtOH % and L/S mL g^−1^) as a function of response variables (*E_y_*, TPC, TPT, DPPH, and ABTS) for the extraction of bioactive compounds from brown algae, with results presented in Table 3.

The highest yield and TPC values for the brown algae extract recovered by MAE was provided by assay MAE11 (60 °C, water, and 40 mL g^−1^), at medium temperature, and highest solvent polarity (water) and L/S ratio. These results can be attributed to water’s solubilization capacity, dissolving various polar compounds (phenolic group) at moderate temperatures [33]. Similarly, the best TPT performance, at lower L/S ratio, was observed for assay MAE9 (60 °C, water and 10 mL g^−1^), while the best antioxidant activities, according to DPPH and ABTS methods, were provided by MAE2 (80 °C, water and 25 mL g^−1^) and MAE8 (80 °C, 50% EtOH, 40 mL g^−1^), respectively. These results indicate that the parameters T, EtOH% and L/S directly influence the recovery of bioactive compounds from brown algae. In addition, the antioxidant potential of brown algae extracts has been recently investigated and associated with the presence of higher amounts of polyphenols, compared to red and green algae extracts [9,34].

**Figure 1:**
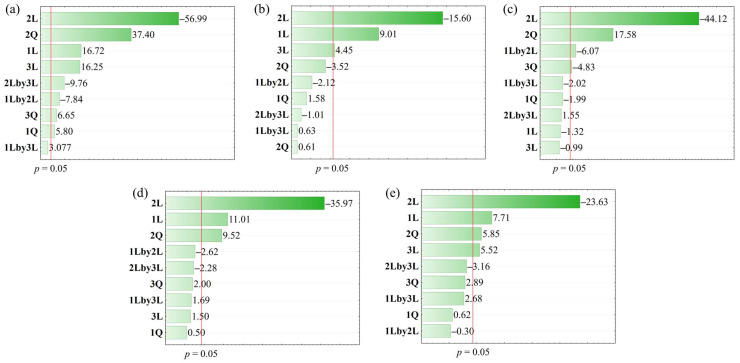
Pareto chart presenting the effect of parameters of MAE in responses (**a**) *E_y_*, (**b**) TPC, (**c**) TPTs, (**d**) DPPH, and (**e**) ABTS. (1) T (°C), (2) EtOH (%), (3) L/S (mL mg^−1^). L—Linear effect, and Q—Quadratic effect.

**Figure 2:**
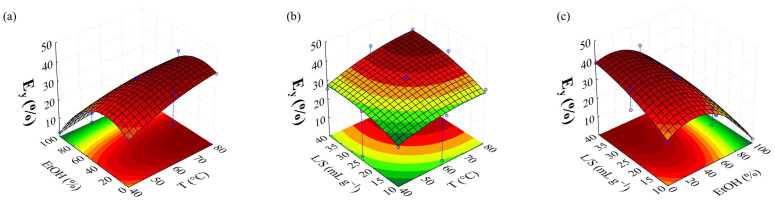
Response surface presenting the effect of parameters of MAE in Ey, where (**a**–**c**) represent the interaction between ethanol/water concentration vs. temperature, liquid/solid ratio vs. temperature, and liquid/solid ratio vs. ethanol/water concentration, respectively.

The Pareto chart was constructed to observe the significant effect of the independent variables on the MAE responses for brown algae (Figure 1). After removing non-significant parameters, the variance analysis (ANOVA, Table S1) shows the proposed regression models (Table 4) with values above 87% of response. A non-significant lack of fit validates the model. The model fits the experimental data, providing the relations between independent and dependent variables for each response studied. The response surfaces methodology (RSM) was also used to define the interactions between these variables, indicating the optimal conditions for each response. Figure 2 presents the response surfaces generated by MAE optimization using BBD for the *E_y_*.

From the Pareto chart (Figure 1a), it is noticeable that 2L, 2Q, 1L, and 3L present the highest significant level (*p* < 0.05) of *E_y_* (Table 4), with an R^2^ regression model of 0.9934, as provided by analysis of variance (ANOVA, Table S1). A non-significant lack of fit validates the model. The EtOH% (2L) showed a negative linear effect, meaning that higher water concentrations improved the extraction yield of brown algae polar compounds within the studied parameters. The interaction effects of the variables were examined using response surface methodology (RSM) by fixing one variable at a medium level. A positive quadratic effect (2Q) suggests an optimal independent variable value that maximizes the response. This relationship is depicted as a parabolic curve with upward concavity (Figure 2a,c). As reported by Kadam et al. [35], higher yield values are due to the abundance of polysaccharides in brown algae which are soluble in strongly polar solvents, such as ethanol/water mixtures with low ethanol concentrations.

The linear positive effects of temperature (1L) and liquid/solid ratio (3L) provide an increase in yield at higher levels of temperature and liquid/solid ratio (Figure 2b). However, the interaction between the ethanol/water concentration and the liquid/solid ratio (2Lby3L) had a negative and significant linear effect, indicating that an increase in these parameters considerably reduced E_y_. Similarly, the interaction between temperature and ethanol/water concentration (1Lby2L) exhibits a significant negative linear effect. Additionally, the quadratic effect of the liquid/solid ratio (3Q) and temperature (1Q) are positive, suggesting a parabolic curve with an upward concavity.

**Figure 3:**
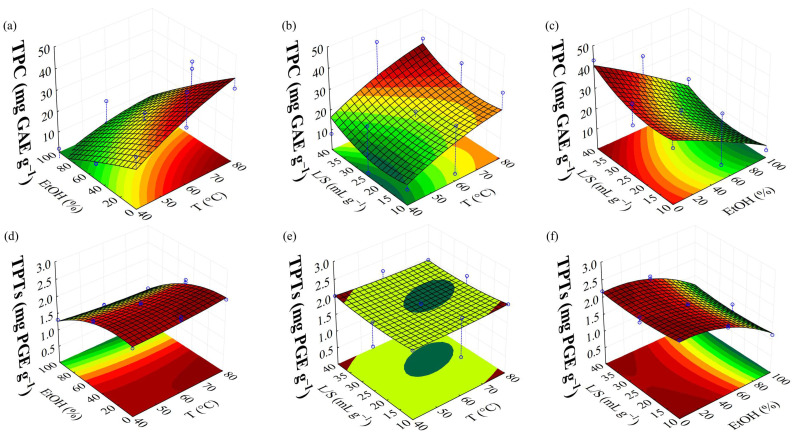
Response surface presenting the effect of parameters of MAE in TPC and TPTs, where (**a**,**d**), (**b**,**e**), and (**c**,**f**) represent the interaction between ethanol/water concentration vs. temperature, liquid/solid ratio vs. temperature, and liquid/solid ratio vs. ethanol/water concentration, respectively.

Figure 3 presents the response surfaces generated by MAE optimization using BBD for the TPC and TPTs. For the TPC Pareto chart (Figure 1b), the first-order linear factors had positive significant effects (*p* < 0.05). The analysis of variance (Table S1) indicated that the proposed regression model for TPC (Table 4) did not present a lack of fit, with variation of 87% (R^2^ = 0.8769). Lower ethanol concentrations are more effective for phenolic recovery (TPC value) from brown algae, with a significant negative linear effect of the ethanol/water concentration (2). Similar behavior was outlined by Korzeniowska et al. [36], who used MAE with water, improving the extraction of phenolic compounds of *Cladophora glomerata*. Conversely, temperature (1L) and liquid/solid ratio (3L) exhibited positive and significant linear effects, i.e., individual increase in these parameters enhances TPC. Higher temperatures increase the dissolution rate by altering the solvent’s properties, contributing to TPC. MAE with water at high temperature helps break the chemical bonds that bind phenolic compounds to the solid matrix, facilitating their release into the solvent solution [37]. Additionally, a higher liquid/solid ratio improves surface contact between the material and the solvent, improving the compounds’ dissolution [8]. The response surfaces (Figure 3a–c) indicate that the highest TPC occurs at high temperatures, low ethanol concentrations, and higher liquid/solid ratios. Magnusson et al. [33], optimizing MAE parameters to recover polyphenols from *Carpophyllum flexuosum*, observed that higher liquid/solid ratio (40:1) and water as solvent improved polyphenol recovery.

The TPTs Pareto chart (Figure 1c) indicates ethanol/water concentration (2L) as the most significant factor, with negative influence, i.e., higher water concentrations enhance TPT values. Also, the quadratic factor (2Q) was positively significant, providing a concave upward parabola (Figure 3d,f), with high TPT at high or low levels of the factors. The interaction between temperature and ethanol/water concentration (1L by 2L) had a negative effect, meaning optimal TPT is achieved when one parameter is high, and the other is low (Figure 3d). Therefore, high temperature with low ethanol/water concentration or low temperature with high ethanol/water concentration enhances TPT. The liquid/solid ratio (3Q) also presented a positive and significant quadratic effect, indicating a parabolic surface with upward concavity (Figure 3e), suggesting that higher concentrations of TPTs are recovered only at high or low levels. The regression model proposed (Table 4) for TPTs did not show a lack of fit, with an explained variation of 99% (R^2^ = 0.9912), according to the analysis of variance (ANOVA) (Table S1).

**Figure 4:**
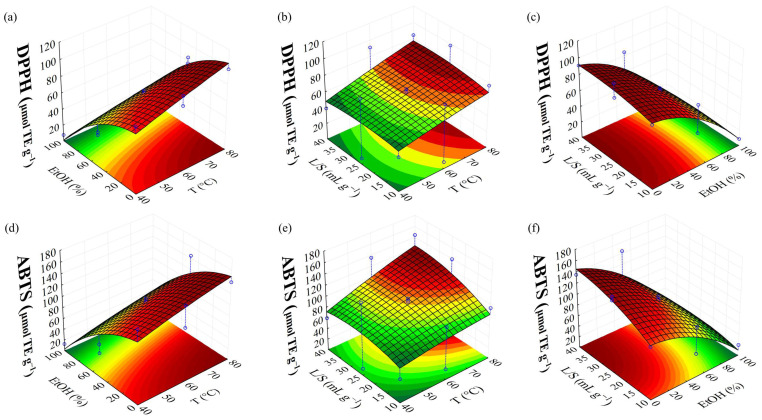
Response surface presenting the effect of parameters of MAE in DPPH and ABTS, where (**a**,**d**), (**b**,**e**), and (**c**,**f**) represent the interaction between ethanol/water concentration vs. temperature, liquid/solid ratio vs. temperature, and liquid/solid ratio vs. ethanol/water concentration, respectively.

Figure 4 presents the response surfaces generated by MAE optimization using BBD for DPPH and ABTS. The Pareto chart of antioxidant capacity (DPPH and ABTS) (Figure 1d,e) shows the negative linear effect of the ethanol/water concentration (2L), indicating that lower ethanol concentrations enhance antioxidant recovery from brown algae. The positive quadratic effect (2Q) suggests optimal concentration that maximizes recovery, forming an upward-facing parabolic curve (Figure 4a,c,d,f). Additionally, the positive linear effect of temperature (2L) indicates that higher temperatures improve antioxidant recovery from brown algae, within the studied range (Figure 4b,d). The liquid/solid ratio shows a significant and positive effect for ABTS (Figure 4e,f), indicating that increasing this parameter enhances antioxidant potential measured by the ABTS reagent. The analysis of variance (Table S1) indicated that the proposed regression model for DPPH and ABTS (Table 4) did not present a lack of fit, with an explained variation of about 96 and 92% (R^2^ = 0.9668 and 0.9252), respectively.

**Figure 5:**
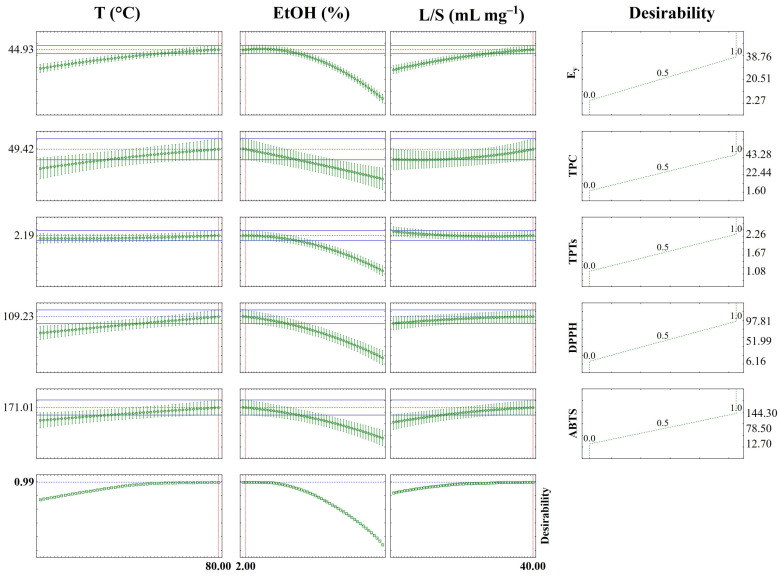
Predicted values of the desirability function of *E_y_*, TPC, TPTs, DPPH, and ABTS of brown algae (*Saccharina latissima*) extracts obtained by MAE.

The desirability function used for process optimization aimed to identify the conditions that provide high yield and an efficient recovery of bioactive compounds, benefiting all responses simultaneously. A global function was created from three regression models and maximized on a scale from 0 (undesirable) to 1 (very desirable) for each factor. Figure 5 illustrates the resulting desirability profile and Figure S2 shows the desirability response surface plots.

The results of the desirability function show that the best MAE performance (high yield and best recovery of bioactive compounds from brown algae) was at 80 °C, 2% EtOH, and 40 mL g^−1^, within the parameters studied (desirability value of 0.99). A new set of experiments was conducted using the optimized conditions, and the experimental values (EV) obtained were compared with the predicted values (PV) for each response, as shown in Table 5. The strong agreement, with a relative error (E%) lower than 4%, demonstrates that the predictive model effectively describes the performance of the MAE of brown algae, considering the evaluated responses.

The optimized conditions were then used to provide continuity to the work, evaluating the quality (properties) of the extract recovered by MAE at the optimized conditions (Section 3.4 and Section 3.5).

### 3.3. PLE and CRE Methods

Table 6 shows the results obtained for the PLE and CRE methods applied for the brown algae (*Saccharina latissima*) using ethanol and water in different concentrations as solvents. The assays focused on the responses of process yield (*E_y_*) and extract quality (concentration of TPC and TPTs and the antioxidant capacity by DPPH and ABTS methods). PLE with water at 120 °C and 80 °C provided the highest E_y_ values, 45.69 ± 1.04 and 36.60 ± 0.43, respectively. Therefore, an increase in PLE temperature (from 40 °C to 120 °C) significantly improves *E_y_* (*p* < 0.05). PLE provided the best yield results compared to CRE, for all conditions evaluated (considering the same solvents). Lower ethanol concentrations resulted in higher *E_y_* for PLE and CRE methods. Sánchez-Camargo et al. [38] reported high extraction yield (*E_y_* = 42.3%) using PLE with water at 125 °C to obtain bioactive compounds from *Sargassum muticum*.

As can be seen in Table 6, PLE from brown algae at 80 °C with water provided the highest TPC and TPT values, of 50.07 ± 1.93 mg GAE g^−1^ and 2.53 ± 0.15 mg PGE g^−1^, respectively, suggesting maximum extraction of phenolic and phlorotannin compounds. Increasing PLE temperature (from 40 °C to 80 °C), with water as solvent, significantly increases the TPC and TPT (*p* < 0.05). PLE at 120 °C provided significantly lower TPC and TPT values compared to the sample recovered at 80 °C, suggesting that excessively high temperatures may have contributed to the degradation of heat-sensitive compounds. The PLE and CRE results show that water was the most effective solvent for phenolics’ recovery. Keramane et al. [9] reported TPC and TPTs values of 49.82 ± 1.40 mg GAE g^−1^ and 2.93 ± 0.08 mg PGE g^−1^, respectively, for PLE (120 °C and ethanol/water 75:25 (*v*/*v*)) from brown algae (*Padina pavonia*), results close to what the present study found for *Saccharina latissima*, highlighting the potential of PLE and validating the parameters selected for the recovery of valuable compounds.

The best conditions for antioxidant recovery from brown algae were PLE at 80 °C with water as solvent (159.10 ± 3.42 μmol TE g^−1^ and ABTS of 224.78 ± 1.80 μmol TE g^−1^). Similar to TPC and TPT, an increase in temperature up to 120 °C (PLE with water) reduced the antioxidant capacity of the extract, suggesting a potential degradation of antioxidant compounds. Nevertheless, the best performance of the water extracts in the DPPH and ABTS assays highlights the water-solubilization of antioxidant compounds. The antioxidant capacity of a brown algae (*Halopteris scoparia*) extract obtained by PLE (120 °C and a mixture of ethanol/water of 75:25) was also reported by Keramane et al. [9], presenting a concentration of 480 ± 26 μmol TE g^−1^ (TEAC).

Environmental factors such as seasonality, geographic location, plant age, and growing conditions, including water temperature, nutrient availability, and exposure to sunlight, can significantly influence the chemical composition of brown algae. These factors can directly affect the concentration of bioactive compounds, impacting the efficiency of extraction processes and the yields obtained [2]. However, natural variations between species will be considered when interpreting the results, as they can directly influence the composition of brown algae.

### 3.4. Comparison of MAE, PLE and CRE Extraction Efficiency

Figure S3 shows the increase in extraction yield and potential of bioactive compounds (TPC, TPTs, and antioxidants (DPPH and ABTS)), using the MAE (optimized) and PLE extraction techniques, compared to the conventional reflux extraction (CRE) method. It is possible to observe an increase in the extract yield using the PLE1 (30 min), PLE2 (30 min), and MAE (optimized) (8 min) techniques compared to CRE (6 h), with values of 5.53%, 37.36%, and 65.76%, respectively. As can be observed, for PLE2 with water at 80 °C the response values for TPC, TPTs, and antioxidant capacity (DPPH and ABTS) were 61.88%, 18.35%, 72.38%, and 80.39% higher than obtained by CRE sampling, respectively. Using MAE (optimized), the values for TPC, DPPH and ABTS increased by 53.90%, 19.18% and 36.42%, respectively. Therefore, both processes can be considered a good alternative to conventional CRE extraction since they provide better results with lower solvent and energy consumption and much shorter extraction times.

Concerning the identification of phenolic compounds using UHPLC q-TOF MS/MS, a number of compounds could be identified in optimized samples (PLE1, PLE2 and optimized MAE) after applying data mining strategies and extracting ion chromatogram of the exact monoisotopic mass adducts ([M − H]^−^, [M + Cl]^−^, [M – H + FA]^−^) of a large list of phenolic compounds found in algae [39,40]. Although we were able to confirm the presence of phenolic compounds using classic quantification methods (Section 3.2 and Section 3.3), its tentative identification by UHPLC coupled to a high-resolution mass spectrometric technique was not possible due to, on one hand, the complexity of phenolics in *S. latissima* brown algae and, on the other hand, its coelution with complex carbohydrates, that implied an unexpected and difficult to overcome matrix effect. Considering the presence of phenolic compounds (measured as TPC and TPT) and the available literature, we can infer that the fraction of phenolics recovered from the macroalgae may be mainly composed of complex components with high molecular weight (bound phenolics or with a high degree of polymerization, such as the phlorotannins and fucoidans). For instance, a very recent study reported by Peng et al. [41] confirmed for the first time the presence of 42 phenolics in the bound fraction of 11 different seaweed species (unfortunately, *S. latissima* was not investigated). Bound phenolics are usually found in organisms interacting with other macromolecules (pectin, cellulose, lignin, protein, etc.) by covalent bonds (in primary cell walls) and ionic bonds (with other food matrixes) [42], making their identification a difficult task. In addition, due to the much higher concentration of carbohydrates (Table 1—Section 3.1) compared to the phenolic compounds, this must have led to a masked separation of the latter, causing a matrix effect that hindered the detection of the compounds of interest. The commonly reported phenolics from macroalgae are usually classified as phenolic acids, flavonoids, stilbenes, bromophenols and phlorotannins [43]. These latter could range from approximately 126.03 g/mol (phloroglucinol monomer) to over 1200 g/mol (as for the case of decamers) [39]. It is worth mentioning the difficulty of identifying this type of phenolics; this explains why studies using high-resolution chromatography-mass spectrometry are very scarce, specifically for *S. latissima*, where quantification studies are usually limited to total phenolic content (TPC) results [9,38]. Therefore, a more complex strategy should be applied in order to obtain a proper phlorotannin identification from *S. latissima*. For instance, Montero et al. [44] successfully characterized phlorotannins from *Cystoseira abies-marina* using a comprehensive 2D LC, elucidating the different polymerization degrees (from 5 to 17 phloroglucinol units). It is important to notice that this characterization strategy required complex and polluting fractionation methodologies due to the high amount of organic toxic solvents employed and, therefore, alternative methods would be needed for the appropriate purification of complex molecules in *S. latissima* and brown algae in general.

### 3.5. Assessment of Oxygen Radical Absorbance Capacity (ORAC)

ORAC assay simulates biological conditions, estimating how antioxidants in brown algae can function within cellular environments to defend against oxidative damage. Based on the previous conditions obtained by analyzing bioactive composts in the extracts obtained by PLE and MAE (Section 3.2 and Section 3.3), the best conditions of PLE and optimized MAE were used to evaluate the capacity to neutralize radicals. Table 7 presents the IC_50_ values for *Saccharina latissima* extracts using different extraction methods compared with ascorbic acid as a positive control.

The extracts obtained by MAE (optimized) show higher antioxidant capacity than PLE1. The PLE2 shows no statistical difference from other samples (*p* < 0.05). Nevertheless, all extracts recovered from the brown algae show potential to inhibit radicals; for instance, Mildenberger et al. [45] assessed the antioxidant capacity of *Saccharina latissima* after different ultrasound-assisted treatments with pure water and methanol/H_2_O (4:1), obtaining values 100-fold higher for aqueous methanol extraction (around 1000 μmolTE g^−1^ dried seaweed), thus indicating that the nature of the solvent used has a strong influence on the antioxidant compounds extracted from this macroalga.

## 4. Conclusions

In the present work, we demonstrated that the application of PLE and MAE is a more direct way to obtain TPC, TPTs, and antioxidant capacity (DPPH and ABTS) from the brown macroalgae *S. latissima*, which is a promising source of natural bioactive compounds, especially polyphenols such as phlorotannin. The MAE technique under optimized conditions and the PLE technique under different extraction conditions showed promising results, emphasizing PLE (80 °C, water), which presented the highest levels of total phlorotannin and a significantly higher antioxidant capacity. These methods also effectively use green solvents, suggesting that they can replace less sustainable traditional techniques. Although the exact identification of the compounds was challenged by the presence of substances of different molecular weights, classical total quantification analyses confirmed the bioactive potential of the recovered extracts. Thus, the research highlights the viability of using brown algae as sustainable sources of bioactive compounds, reinforcing the importance of green extraction strategies for exploring marine natural resources. Future studies in cellular models could evaluate the bioactivity of the extracts, investigating their bioavailability, cytotoxicity and therapeutic effects, especially of polyphenols such as phlorotannins.

## Figures and Tables

**Table 1:** Proximal composition of *Saccharina latissima* used in this work.

	*S. latissima*
Moisture (fresh sample) (wt. %)	13.45 ± 0.58
Protein (dwt. %)	6.29 ± 0.02
Lipids (dwt. %)	1.06 ± 0.01
Ash (dwt. %)	9.55 ± 0.08
Carbohydrates * (dwt. %)	69.79 ± 0.74

* Estimated by difference.

**Table 2:** Piecewise model parameters fitted to kinetic extraction of MAE (80 °C, 2% EtOH, and 40 mL g^−1^), PLE 1 (40 °C, 50% EtOH), and PLE2 (80 °C, water).

Parameters	MAE	PLE1	PLE2
*a*_1_ (g)	0.0137	0.0947	0.1549
*k*_1_ (g min^−1)^	0.0639	0.0590	0.0875
*t*CER (min)	2.0620	7.7305	7.6631
*k*_2_ (g min^−1^)	0.0063	0.0141	0.0096
*t*FER (min)	7.0632	25.9994	28.5496
*k*_3_ (g min^−1^)	0.0002	0.0038	0.0022
R^2^	0.9637	0.9840	0.9674

MAE—60 °C, 50% EtOH, and 25 mL g^−1^, PLE1—10 MPa, 4 mL min^−1^ 40 °C, 50% EtOH, PLE2—10 MPa, 4 mL min^−1^, 80 °C, and water.

**Table 3:** Experimental values of MAE yield (*E_y_*) from brown algae (*Saccharina latissima*) obtained by MAE, and quality of the recovered extracts (TPC, TPTs, DPPH and ABTS).

Assays	Real Variables			*E_y_* (%)	TPC (mg GAE g^−1^)	TPTs (mg PGE g^−1^)	DPPH (µmol TE g^−1^)	ABTS (µmol TE g^−1^)
	(1) T (°C)	(2) EtOH (%)	(3) L/S (mL g^−1^)					
MAE1	40	0	25	26.44	24.76	2.01	80.78	132.77
MAE2	80	0	25	38.01	34.94	2.15	97.81	139.33
MAE3	40	100	25	2.28	1.82	1.32	7.61	12.70
MAE4	80	100	25	2.79	1.61	1.09	8.27	15.31
MAE5	40	50	10	20.99	7.78	1.99	42.65	43.65
MAE6	80	50	10	29.46	32.47	2.04	77.22	93.18
MAE7	40	50	40	25.71	8.09	2.04	39.22	59.57
MAE8	80	50	40	38.53	35.87	1.96	84.33	144.30
MAE9	60	0	10	22.55	27.22	2.26	79.06	96.76
MAE10	60	100	10	2.27	3.77	1.14	8.51	19.98
MAE11	60	0	40	38.76	43.28	2.18	90.95	135.35
MAE12	60	100	40	4.70	14.88	1.16	6.17	16.96
MAE13	60	50	25	33.91	21.36	1.87	68.03	103.53
MAE14	60	50	25	32.50	17.66	1.93	61.78	90.40
MAE15	60	50	25	33.31	16.73	1.88	64.96	97.56

**Table 4:** Fitted model equations and determination coefficients of response of MAE analysis.

Response	Regression Equation	R^2^
*E_y_*	21.04 + 4.17(1) + 1.06(1)^2^ − 14.22(2) + 6.87(2)^2^ + 4.05(3) + 1.22(3)^2^ − 2.76(1) * (2) − 3.44(2) * (3)	0.9934
TPC	19.71 + 7.81(1) − 13.52(2) + 3.86(3)	0.8769
TPTs	1.78 − 0.49(2) + 0.14(2)^2^ − 0.04(3)^2^ − 0.09(1) * (2)	0.9912
DPPH	51.88 + 12.17(1) − 39.75(2) + 7.75(2)^2^	0.9668
ABTS	75.82 + 17.93(1) − 54.91(2) + 10.01(2)^2^ + 12.82(3)	0.9252

(1) T (°C), (2) EtOH (%), (3) L/S (mL mg^−1^).

**Table 5:** Comparison of predicted and experimental values for the different responses for the validation of the optimum conditions obtained from the desirability function.

Response Variable	Desirability Conditions			PV	EV	E%
	T (°C)	EtOH (%)	L/S (mL g^−1^)			
*E_y_* (%)	80.00	2.00	40.00	44.93	44.17	1.69
TPC (mg GAE g^−1^)				49.42	47.60	3.68
TPTs (mg PGE g^−1^)				2.19	2.16	1.37
DPPH (μmol TE g^−1^)				109.23	113.50	3.91
ABTS (μmol TE g^−1^)				171.01	170.16	0.49

T (°C): Temperature; EtOH (%): Ethanol/water concentration; L/S (mL g^−1^): Liquid/solid ratio; PV: Predicted value; EV: Experimental value; E%: relative error (%).

**Table 6:** Responses of E_y_, TPC, TPTs, and antioxidant capacity (DPPH and ABTS assays) obtained for the extract samples recovered by PLE and CRE processes, under different conditions, from brown algae (*Saccharina latissima*).

Experimental Conditions	*E_y_* (%)	TPC (mg GAE g^−1^)	TPTs (mg PGE g^−1^)	DPPH (μmol TE g^−1^)	ABTS (μmol TE g^−1^)
PLE—40 °C, 100% EtOH	2.53 ± 0.36 ^e^	6.85 ± 0.93 ^e^	0.83 ± 0.04 ^e^	5.21 ± 0.02 ^e^	14.19 ± 0.55 ^f^
PLE—40 °C, 50% EtOH	28.12 ± 0.92 ^c^	23.83 ± 1.41 ^d^	1.90 ± 0.06 ^cd^	7.07 ± 0.08 ^e^	35.65 ± 0.79 ^d^
PLE—40 °C, Water	26.33 ± 0.67 ^c^	31.85 ± 2.45 ^bc^	2.07 ± 0.13 ^bc^	84.21 ± 0.97 ^d^	209.55 ± 5.54 ^b^
PLE—80 °C, Water	36.60 ± 0.43 ^b^	50.07 ± 1.93 ^a^	2.53 ± 0.15 ^a^	159.10 ± 3.42 ^a^	224.78 ± 1.80 ^a^
PLE—120 °C, Water	45.69 ± 1.04 ^a^	36.79 ± 2.83 ^b^	1.93 ± 0.16 ^c^	135.32 ± 0.37 ^b^	131.57 ± 1.24 ^c^
CRE—100% EtOH	3.14 ± 0.10 ^e^	5.62 ± 0.53 ^e^	0.84 ± 0.03 ^e^	6.07 ± 0.28 ^e^	13.08 ± 0.62 ^f^
CRE—50% EtOH	24.17 ± 0.70 ^d^	21.98 ± 1.07 ^d^	1.64 ± 0.15 ^d^	9.12 ± 0.22 ^e^	25.27 ± 0.23 ^e^
CRE—Water	26.65 ± 0.41 ^c^	30.93 ± 1.85 ^c^	2.22 ± 0.06 ^b^	92.30 ± 2.24 ^c^	124.61 ± 3.63 ^c^

Samples identified with the same letter in the column did not present a significant difference (*p* < 0.05) in the Tukey test.

**Table 7:** ORAC values (IC_50_) for extracts from *Saccharina latissima*.

Extractions	ORAC IC_50_ (µg extract/mL) *
PLE1	19.04 ± 0.92 ^a^
PLE2	15.33 ± 2.19 ^ab^
Optimized MAE	13.89 ± 0.39 ^b^
Ascorbic Acid (positive control)	0.48 ± 0.03 ^c^

* IC_50_ means that share the same letter do not present any significant difference at a confidence level of 95%. Conditions: PLE1—(40 °C, 50% EtOH), PLE2—(80 °C, Water), and optimized MAE—(80 °C, 2% EtOH, and 40 mL g^−1^).

## Data Availability

The original contributions presented in this study are included in the article/Supplementary Materials. Further inquiries can be directed to the corresponding authors.

## Supplementary Materials

The following supporting information can be downloaded at: https://www.mdpi.com/article/10.3390/foods14061017/s1, Figure S1: Piecewise model parameters fitted to experimental kinetics extraction curves for brown algae by (a) MAE, (b) PLE1 and (c) PLE2; Figure S2: Surface graph of the desirability function of Ey, TPC, TPTs, DPPH, and ABTS of brown algae (*Saccharina latissima*) extracts obtained by MAE. (I), (II), and (III) represent interaction between ethanol/water concentration vs. temperature, liquid/solid ratio vs. temperature, and liquid/solid ratio vs. ethanol/water concentration, respectively; Figure S3: Increased extraction yield and potential of bioactive compounds from MAE (optimized) and PLE extraction techniques compared to CRE. * CRE extraction with water; Optimized MAE—80 °C, 2% EtOH, and 40 mL g^−1^; PLE1—40 °C, 50% EtOH; and PLE2—80 °C, water; Table S1: Analysis of variance (ANOVA) for Ey, TPC, TPTs, DPPH and ABTS obtained by MAE in the experimental design (BBD).

## References

[1] Jang H., Lee J., Park Y.-K., Lee J.-Y. (2024). Exploring the Health Benefits and Concerns of Brown Seaweed Consumption: A Comprehensive Review of Bioactive Compounds in Brown Seaweed and Its Potential Therapeutic Effects. J. Agric. Food Res..

[2] Irianto I., Naryaningsih A., Trisnawati N.W., Astuti A., Komariyah K., Qomariyah L., Chaidir C., Saputri A., Wulandari R., Rizkiyah D.N. (2024). From Sea to Solution: A Review of Green Extraction Approaches for Unlocking the Potential of Brown Algae. S. Afr. J. Chem. Eng..

[3] Lee Z.J., Xie C., Ng K., Suleria H.A.R. (2023). Unraveling the Bioactive Interplay: Seaweed Polysaccharide, Polyphenol and Their Gut Modulation Effect. Crit. Rev. Food Sci. Nutr..

[4] Liu X., Yuan W., Sharma-Shivappa R., van Zanten J. (2017). Antioxidant Activity of Phlorotannins from Brown Algae. Int. J. Agric. Biol. Eng..

[5] Montero L., Sánchez-Camargo A.P., García-Cañas V., Tanniou A., Stiger-Pouvreau V., Russo M., Rastrelli L., Cifuentes A., Herrero M., Ibáñez E. (2016). Anti-Proliferative Activity and Chemical Characterization by Comprehensive Two-Dimensional Liquid Chromatography Coupled to Mass Spectrometry of Phlorotannins from the Brown Macroalga *Sargassum muticum* Collected on North-Atlantic Coasts. J. Chromatogr. A.

[6] Abdelhamid A., Lajili S., Elkaibi M.A., Ben Salem Y., Abdelhamid A., Muller C.D., Majdoub H., Kraiem J., Bouraoui A. (2019). Optimized Extraction, Preliminary Characterization and Evaluation of the in Vitro Anticancer Activity of Phlorotannin-Rich Fraction from the Brown Seaweed, Cystoseira Sedoides. J. Aquat. Food Product. Technol..

[7] Sardari R.R.R., Prothmann J., Gregersen O., Turner C., Nordberg Karlsson E. (2020). Identification of Phlorotannins in the Brown Algae, *Saccharina latissima* and *Ascophyllum nodosum* by Ultra-High-Performance Liquid Chromatography Coupled to High-Resolution Tandem Mass Spectrometry. Molecules.

[8] Toan T.Q., Phong T.D., Tien D.D., Linh N.M., Mai Anh N.T., Hong Minh P.T., Duy L.X., Nghi D.H., Pham Thi H.H., Nhut P.T. (2021). Optimization of Microwave-Assisted Extraction of Phlorotannin from Sargassum Swartzii (Turn.) C. Ag. with Ethanol/Water. Nat. Prod. Commun..

[9] Keramane B., Sánchez-Camargo A.d.P., Montero L., Laincer F., Bedjou F., Ibañez E. (2023). Pressurized Liquid Extraction of Bioactive Extracts with Antioxidant and Antibacterial Activity from Green, Red and Brown Algerian Algae. Algal Res..

[10] Sadeghi A., Rajabiyan A., Nabizade N., Meygoli Nezhad N., Zarei-Ahmady A. (2024). Seaweed-Derived Phenolic Compounds as Diverse Bioactive Molecules: A Review on Identification, Application, Extraction and Purification Strategies. Int. J. Biol. Macromol..

[11] Sánchez-Camargo A.P., Montero L., Cifuentes A., Herrero M., Ibáñez E. (2016). Application of Hansen Solubility Approach for the Subcritical and Supercritical Selective Extraction of Phlorotannins from Cystoseira Abies-Marina. RSC Adv..

[12] He Z., Chen Y., Chen Y., Liu H., Yuan G., Fan Y., Chen K. (2013). Optimization of the Microwave-Assisted Extraction of Phlorotannins from *Saccharina japonica* Aresch and Evaluation of the Inhibitory Effects of Phlorotannin-Containing Extracts on HepG2 Cancer Cells. Chin. J. Oceanol. Limnol..

[13] (1997). AOAC Methods 970.22, 972.15, 963.15. Official Methods of Analysis of the Association of Official Analytical Chemistry.

[14] (2005). AOAC Method 920.39C. Hods of Analysis of the Association of Official Analytical Chemists.

[15] Rudke A.R., da Silva M., de Andrade C.J., Vitali L., Ferreira S.R.S. (2022). Green Extraction of Phenolic Compounds and Carrageenan from the Red Alga Kappaphycus Alvarezii. Algal Res..

[16] Gonçalves Rodrigues L.G., Mazzutti S., Vitali L., Micke G.A., Ferreira S.R.S. (2019). Recovery of Bioactive Phenolic Compounds from Papaya Seeds Agroindustrial Residue Using Subcritical Water Extraction. Biocatal. Agric. Biotechnol..

[17] Weinhold T.d.S., Bresciani L.F.V., Tridapalli C.W., Yunes R.A., Hense H., Ferreira S.R.S. (2008). *Polygala cyparissias* Oleoresin: Comparing CO_2_ and Classical Organic Solvent Extractions. Chem. Eng. Process. Process Intensif..

[18] Singleton V.L., Orthofer R., Lamuela-Raventós R.M. (1999). Analysis of Total Phenols and Other Oxidation Substrates and Antioxidants by Means of Folin-Ciocalteu Reagent. Methods Enzymol..

[19] dos Santos L.C., Mendiola J.A., Sánchez-Camargo A.d.P., Álvarez-Rivera G., Viganó J., Cifuentes A., Ibáñez E., Martínez J. (2021). Selective Extraction of Piceatannol from *Passiflora edulis* By-Products: Application of HSPs Strategy and Inhibition of Neurodegenerative Enzymes. Int. J. Mol. Sci..

[20] Lopes G., Sousa C., Silva L.R., Pinto E., Andrade P.B., Bernardo J., Mouga T., Valentão P. (2012). Can Phlorotannins Purified Extracts Constitute a Novel Pharmacological Alternative for Microbial Infections with Associated Inflammatory Conditions?. PLoS ONE.

[21] Mensor L.L., Menezes F.S., Leitão G.G., Reis A.S., dos Santos T.C., Coube C.S., Leitão S.G. (2001). Screening of Brazilian Plant Extracts for Antioxidant Activity by the Use of DPPH Free Radical Method. Phytother. Res..

[22] Re R., Pellegrini N., Proteggente A., Pannala A., Yang M., Rice-Evans C. (1999). Antioxidant Activity Applying an Improved ABTS Radical Cation Decolorization Assay. Free Radic. Biol. Med..

[23] Ou B., Hampsch-Woodill M., Prior R.L. (2001). Development and Validation of an Improved Oxygen Radical Absorbance Capacity Assay Using Fluorescein as the Fluorescent Probe. J. Agric. Food Chem..

[24] Michalak L., Morales-Lange B., Montero R., Horn S.J., Mydland L.T., Øverland M. (2023). Impact of Biorefinery Processing Conditions on the Bioactive Properties of Fucoidan Extracts from Saccharina Latissima on SHK-1 Cells. Algal Res..

[25] Rhein-Knudsen N., Reyes-Weiss D., Horn S.J. (2023). Extraction of High Purity Fucoidans from Brown Seaweeds Using Cellulases and Alginate Lyases. Int. J. Biol. Macromol..

[26] Reyes B.A.S., Dufourt E.C., Ross J., Warner M.J., Tanquilut N.C., Leung A.B. (2018). Selected Phyto and Marine Bioactive Compounds: Alternatives for the Treatment of Type 2 Diabetes. Stud. Nat. Prod. Chem..

[27] Sovová H. (1994). Rate of the Vegetable Oil Extraction with Supercritical CO_2_—I. Modelling of Extraction Curves. Chem. Eng. Sci..

[28] Rodrigues L.G.G., Mazzutti S., Siddique I., da Silva M., Vitali L., Ferreira S.R.S. (2020). Subcritical Water Extraction and Microwave-Assisted Extraction Applied for the Recovery of Bioactive Components from Chaya (*Cnidoscolus aconitifolius* Mill.). J. Supercrit. Fluids.

[29] Rudke A.R., Mazzutti S., Andrade K.S., Vitali L., Ferreira S.R.S. (2019). Optimization of Green PLE Method Applied for the Recovery of Antioxidant Compounds from Buriti (*Mauritia flexuosa* L.) Shell. Food Chem..

[30] Murugesh C.S., Rastogi N.K., Subramanian R. (2018). Athermal Extraction of Green Tea: Optimisation and Kinetics of Extraction of Polyphenolic Compounds. Innov. Food Sci. Emerg. Technol..

[31] Mezzomo N., Martínez J., Ferreira S.R.S. (2009). Supercritical Fluid Extraction of Peach (*Prunus persica*) Almond Oil: Kinetics, Mathematical Modeling and Scale-Up. J. Supercrit. Fluids.

[32] Ferreira S.R.S., Meireles M.A.A. (2002). Modeling the Supercritical Fluid Extraction of Black Pepper (*Piper Nigrum* L.) Essential Oil. J. Food Eng..

[33] Magnusson M., Yuen A.K.L., Zhang R., Wright J.T., Taylor R.B., Maschmeyer T., de Nys R. (2017). A Comparative Assessment of Microwave Assisted (MAE) and Conventional Solid-Liquid (SLE) Techniques for the Extraction of Phloroglucinol from Brown Seaweed. Algal Res..

[34] Yuan Y., Zhang J., Fan J., Clark J., Shen P., Li Y., Zhang C. (2018). Microwave Assisted Extraction of Phenolic Compounds from Four Economic Brown Macroalgae Species and Evaluation of Their Antioxidant Activities and Inhibitory Effects on α-Amylase, α-Glucosidase, Pancreatic Lipase and Tyrosinase. Food Res. Int..

[35] Kadam S.U., Tiwari B.K., Smyth T.J., O’Donnell C.P. (2015). Optimization of Ultrasound Assisted Extraction of Bioactive Components from Brown Seaweed Ascophyllum Nodosum Using Response Surface Methodology. Ultrason. Sonochem.

[36] Korzeniowska K., Łęska B., Wieczorek P.P. (2020). Isolation and Determination of Phenolic Compounds from Freshwater Cladophora Glomerata. Algal Res..

[37] Quiles-Carrillo L., Mellinas C., Garrigos M.C., Balart R., Torres-Giner S. (2019). Optimization of Microwave-Assisted Extraction of Phenolic Compounds with Antioxidant Activity from Carob Pods. Food Anal. Methods.

[38] Sánchez-Camargo A.d.P., Montero L.A., Stiger-Pouvreau V., Tanniou A., Cifuentes A., Herrero M., Ibáñez E. (2016). Considerations on the Use of Enzyme-Assisted Extraction in Combination with Pressurized Liquids to Recover Bioactive Compounds from Algae. Food Chem..

[39] Erpel F., Mateos R., Pérez-Jiménez J., Pérez-Correa J.R. (2020). Phlorotannins: From Isolation and Structural Characterization, to the Evaluation of Their Antidiabetic and Anticancer Potential. Food Res. Int..

[40] Goksen G. (2023). Elucidation and Quantification Health-Promoting Phenolic Compounds, Antioxidant Properties and Sugar Levels of Ultrasound Assisted Extraction, Aroma Compositions and Amino Acids Profiles of Macroalgae, Laurencia Papillosa. Ultrason. Sonochem.

[41] Peng Z., Wu Y., Fu Q., Xiao J. (2024). Free and Bound Phenolic Profiles and Antioxidant Ability of Eleven Marine Macroalgae from the South China Sea. Front. Nutr..

[42] Wang Z., Li S., Ge S., Lin S. (2020). Review of Distribution, Extraction Methods, and Health Benefits of Bound Phenolics in Food Plants. J. Agric. Food Chem..

[43] Agregán R., Munekata P.E.S., Franco D., Dominguez R., Carballo J., Lorenzo J.M. (2017). Phenolic Compounds from Three Brown Seaweed Species Using LC-DAD–ESI-MS/MS. Food Res. Int..

[44] Montero L., Herrero M., Ibáñez E., Cifuentes A. (2014). Separation and Characterization of Phlorotannins from Brown Algae Cystoseira Abies-marina by Comprehensive Two-Dimensional Liquid Chromatography. Electrophoresis.

[45] Mildenberger J., Stangeland J.K., Rebours C. (2022). Antioxidative Activities, Phenolic Compounds and Marine Food Allergens in the Macroalgae *Saccharina latissima* Produced in Integrated Multi-Trophic Aquaculture Systems. Aquaculture.

