# Supplementary material for: Green Extraction Methods Applied to the Brown Macroalga Saccharina latissima: Assessing Yield, Total Phenolics, Phlorotannins and Antioxidant Capacity
Source: Foods. 2025 Mar 17;14(6):1017. doi: 10.3390/foods14061017 (PMC11941721; doi:10.3390/foods14061017)
Supplement: Supplementary file 1 [file foods-14-01017-s001.zip › foods-3468642-supplementary.pdf]

Supplementary material

Figure S1. Piecewise model parameters fitted to experimental kinetics extraction curves for brown algae by a) MAE, b) PLE1 and c) PLE2.

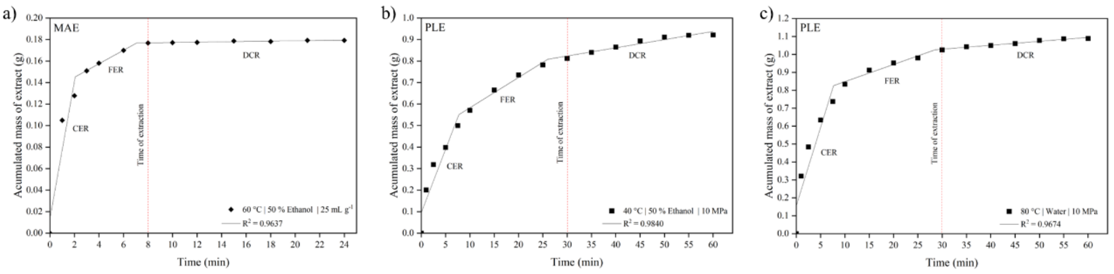

*MAE - 60 ºC, 50 % EtOH, and 25 mL g-1, PLE1 - 10 MPa, 4 mL min-1 40 °C, 50% EtOH, PLE2 - 10 MPa, 4 mL min-1, 80 °C, and 0% EtOH (water).

Figure S2. Surface graph of the desirability function of E_y, TPC, TPTs, DPPH, and ABTS of brown algae (*Saccharina latissima*) extracts obtained by MAE. (I), (II), and (III) represent interaction between ethanol/water concentration vs. temperature, liquid/ solid ratio vs. temperature, and liquid/solid ratio vs. ethanol/ water concentration, respectively.

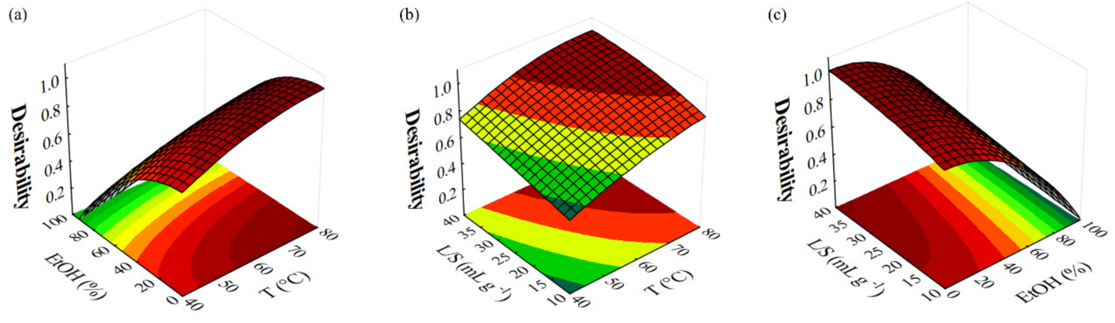

Figure S3. Increased extraction yield and potential of bioactive compounds from MAE (optimized) and PLE extraction techniques compared to CRE. * CRE extraction with water; Optimized MAE - 80 °C, 2% EtOH, and 40 mL g-1; PLE1 - 40 °C, 50% EtOH); and PLE2 - 80 °C, Water.

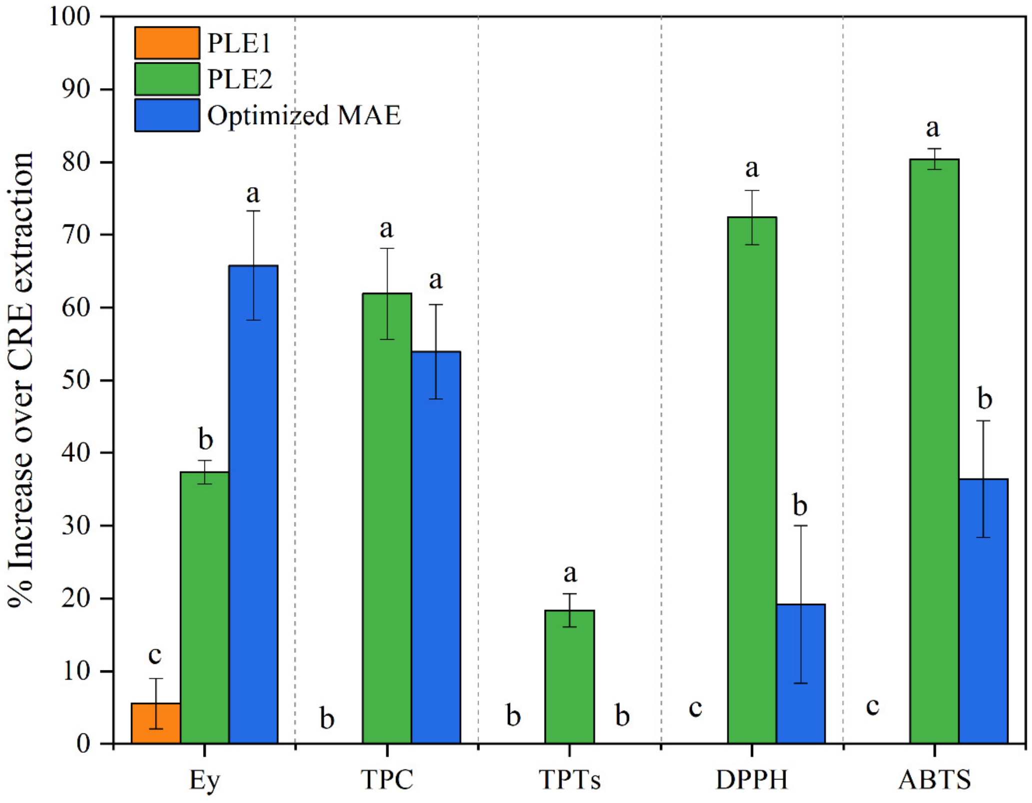

*Columns with the same letters for each analysis did not present a significant difference (p<0.05) in the Tukey test

Table S1. Analysis of variance (ANOVA) for E_y, TPC, TPTs, DPPH and ABTS obtained by MAE in the experimental design (BBD).

**E_y**	SS	df	MS	F	p
T (L)	139.160	1	139.1600	279.598	0.004*
T (Q)	16.719	1	16.7190	33.591	0.029*
EtOH (L)	1616.747	1	1616.7470	3248.343	0.000*
EtOH (Q)	696.259	1	696.2590	1398.914	0.001*
L/S (L)	131.399	1	131.3990	264.005	0.004*
L/S (Q)	22.029	1	22.0290	44.261	0.022*
T by EtOH	30.565	1	30.5650	61.410	0.016*
T by L/S	4.712	1	4.7120	9.467	0.091
EtOH by L/S	47.451	1	47.4510	95.338	0.010*
Lack of Fit	16.728	3	5.5760	11.203	0.083
Pure Error	0.995	2	0.4980		
Total SS	2694.685	14			
**TPC**	SS	df	MS	F	p
T (L)	487.407	1	487.4070	81.201	0.012*
T (Q)	15.035	1	15.0350	2.505	0.254
EtOH (L)	1461.439	1	1461.4390	243.472	0.004*
EtOH (Q)	2.265	1	2.2650	0.377	0.602
L/S (L)	119.097	1	119.0970	19.841	0.047*
L/S (Q)	74.348	1	74.3480	12.386	0.072
T by EtOH	27.051	1	27.0510	4.507	0.168
T by L/S	2.382	1	2.3820	0.397	0.593
EtOH by L/S	6.098	1	6.0980	1.016	0.420
Lack of Fit	297.049	3	99.0160	16.496	0.058
Pure Error	12.005	2	6.0020		
Total SS	2511.056	14			
**TPTs**	SS	df	MS	F	p
T (L)	0.002	1	0.0017	1.742	0.318
T (Q)	0.004	1	0.0039	3.970	0.185
EtOH (L)	1.890	1	1.8901	1945.173	0.001*
EtOH (Q)	0.300	1	0.3005	309.203	0.003*
L/S (L)	0.001	1	0.0010	0.980	0.427
L/S (Q)	0.023	1	0.0227	23.347	0.040*
T by EtOH	0.036	1	0.0358	36.798	0.026*
T by L/S	0.004	1	0.0040	4.089	0.181
EtOH by L/S	0.002	1	0.0024	2.419	0.260
Lack of Fit	0.018	3	0.0061	6.315	0.140
Pure Error	0.002	2	0.0010		
Total SS	2.301	14			
**DPPH**	SS	df	MS	F	p

T (L)	0.00119	1	0.00119	121.298	0.008*
T (Q)	0.00000	1	0.00000	0.247	0.668
EtOH (L)	0.01264	1	0.01264	1293.980	0.001*
EtOH (Q)	0.00089	1	0.00089	90.729	0.011*
L/S (L)	0.00002	1	0.00002	2.238	0.273
L/S (Q)	0.00004	1	0.00004	4.008	0.183
T by EtOH	0.00007	1	0.00007	6.861	0.120
T by L/S	0.00003	1	0.00003	2.843	0.234
EtOH by L/S	0.00005	1	0.00005	5.182	0.151
Lack of Fit	0.00049	3	0.00016	16.757	0.057
Pure Error	0.00002	2	0.00001		
Total SS	0.01541	14			
**ABTS**	SS	df	MS	F	p
T (L)	0.00257	1	0.00257	59.522	0.016*
T (Q)	0.00002	1	0.00002	0.381	0.600
EtOH (L)	0.02412	1	0.02412	558.265	0.002*
EtOH (Q)	0.00148	1	0.00148	34.270	0.028*
L/S (L)	0.00132	1	0.00132	30.454	0.031*
L/S (Q)	0.00036	1	0.00036	8.331	0.102*
T by EtOH	0.00000	1	0.00000	0.091	0.792
T by L/S	0.00031	1	0.00031	7.172	0.116
EtOH by L/S	0.00043	1	0.00043	10.019	0.087
Lack of Fit	0.00238	3	0.00079	18.354	0.052
Pure Error	0.00009	2	0.00004		
Total SS	0.03296	14			

*Significant parameters
